# The Prevalence of Root Sensitivity following Periodontal Therapy: A Systematic Review

**DOI:** 10.1155/2012/407023

**Published:** 2012-10-31

**Authors:** Y. H. Lin, D. G. Gillam

**Affiliations:** Centre for Adult Oral Health, Institute of Dentistry, Barts and the London School of Medicine and Dentistry, Queen Mary University, London E1 2AD, UK

## Abstract

The reported prevalence of dentine/root (hyper)sensitivity (DH/RS) in the published literature varies, and this may be due in part to a) the different study populations and (b) the different methodologies employed in evaluating the pain response. According to von Troil et al. (2002) there are limited data available in terms of the prevalence and intensity of DH/RS following periodontal therapy. *Objectives*. The aim of the present study was therefore to review the literature in order to identify all relevant studies for inclusion and to determine whether there was any evidence of DH/RS following periodontal procedures in the published literature up to 31st December 2009 using an agreed search protocol. *Methods*. 840 papers were identified, from searching both electronic databases (PUBMED) and hand searching of relevant written journals. Twelve papers were subsequently accepted for inclusion. *Results*. The results of the present study would indicate that the reported prevalence for DH/RS (following nonsurgical therapy) was between 62.5% and 90% one day after treatment decreasing to approximately 52.6% to 55% after one week. The prevalence of DH/RS following surgical therapy was between 76.8% and 80.4% one day after treatment subsequently decreasing over time to 36.8% after 1 week, 33.4% after 2 weeks, 29.6% after 4 weeks, and 21.7% after 8 weeks. *Conclusions*. It is evident from reviewing the included studies that patients may suffer from mild discomfort following periodontal procedures although both the prevalence and intensity of DH/RS may vary depending on the duration and the type of procedure involved. Most of the studies included in this paper would tend to suggest that DH/RS may be relatively mild/moderate in nature and transient in duration.

## 1. Introduction

 According to Addy et al. [1] and a Canadian Consensus Document [2], dentine hypersensitivity (DH) can be defined as “pain derived from exposed dentine in response to chemical, thermal, tactile or osmotic stimuli which cannot be explained as arising from any other dental defect or pathology.” This definition was subsequently modified by the Canadian Board on DH [2] which suggested that “disease” is a more appropriate term than “pathology.” From the literature it was apparent that there are other terms used to describe this clinical condition, for example, cervical dentine sensitivity (CDS) or cervical dentine hypersensitivity (CDH) or dentine sensitivity (DS), and root dentine sensitivity (RDS)/root dentine hypersensitivity (RDH). To date, the term “dentine hypersensitivity” (DH) has been preferred in the published literature due in part to its historical significance [3]. Another term “root sensitivity” (RS) was recently suggested by the European Federation of Periodontology [4] to describe tooth sensitivity associated with periodontal disease and its treatment. Epidemiological studies have been undertaken on DH in order to assist our understanding of the prevalence, progression, causative factors, and the preventive or therapeutic measures of the condition. However despite the abundance of papers in the published literature on the condition several researchers have previously reported that there are limited data on its epidemiology and prevalence [5–7]. 

 According to a previous systematic review by von Troil et al. [8] there is also limited data on the prevalence of RS following periodontal therapy. The problem however in reviewing the various published papers to identify the actual prevalence of RS is that historically most if not all of these papers include both subjects complaining of DH and those patients who may be complaining of RS following periodontal therapy [9]. Data from these studies evaluating DH/RS in individuals that have a periodontal condition or have received periodontal treatment in the form of scaling procedures suggest that these individuals may have a higher prevalence than previously reported in the literature (60–98%) [7, 9–15]. Data from the published studies would appear to suggest that DH/RS may affect individuals of all ages, although the peak prevalence was reported to be between the ages of 30 and 60 years [1, 6, 16–22]. Interestingly a higher prevalence figure has been reported in females compared to males, but this does not appear to be statistically significant although numerically more females were reported to have experienced more sensitivity than males [1, 6, 16, 17, 22–24]. This observation however may be due to a number of reasons but generally it is accepted that females have a greater awareness and interest in general and oral health issues [3].

## 2. Aim and Objectives

The objective of this paper was to therefore examine the available literature to determine the prevalence of root sensitivity (RS) following periodontal therapy (nonsurgical and surgical procedure) and to evaluate whether there is any potential causal link between periodontal (nonsurgical and surgical) therapy and root sensitivity.

## 3. Methodology

The search methodology used for the present based on a modified version of von Troll et al. [8] includes both short-term and long-term studies (see below). 

### 3.1. Selection Criteria

#### 3.1.1. Types of Study

This paper included any type of studies in which patients were treated by periodontal therapy and the prevalence or the intensity as assessed by recognized pain scores (e.g., VAS) was evaluated following treatment. 

#### 3.1.2. Types of Subject

Included criteria for relevant studies were dentate, healthy adults (at least 18 years of age) with/without reported periodontitis undergoing periodontal treatment(s). 

Types of outcome measurement are as follows:

1. history of DH/RS as assessed as baseline data,
2. the prevalence or the intensity of DH/RS following periodontal therapy,
3. the methodology used in studies to evaluate root sensitivity (clinical/patient based),
4. the duration of each included study which can be divided as single application, short term (<3 months), moderate term (4–11 months), and long term (>12 months),
5. the treatment intervention used during study (pain relief and desensitizing treatment), and
6. intervention studies—the assessment of the desensitizing products following scaling and/or surgical procedures.

## 4. Search Strategy

The search strategy included using hand searching or electronic databases (PUBMED) up to 31st December 2009. Hand searching also included examining relevant published or incomplete journals. The searching key words in PUBMED were (root OR pulp OR cervical OR tooth OR teeth OR dentin∗ OR dental) AND (sensitiv∗ OR hypersensitiv∗ OR pain∗) AND (periodont∗) AND (random∗ OR trial OR (randomized controlled trial [pt]) OR (controlled clinical trial [pt]) OR cohort∗ OR longitudinal∗ OR “follow up” OR prospective∗ OR case-control). 

## 5. Statistical Analysis

Statistical analysis of data from these studies was not attempted due to the variations in the study design, methodology, study duration, and reporting of the pain response (percentages or VAS scores or pain categories, etc.) following the periodontal procedures.

## 6. Method of the Review

A review of the abstracts and titles was carried out by one of the authors (Y. H. Lin) who then obtained copies of all the relevant studies where available. Two reviewers (Y. H. Lin and D. G. Gillam) sought to determine the eligibility of the papers and data extraction. Any differences as to inclusion or exclusion of papers were resolved following a discussion between Y. H. Lin and D. G. Gillam.

## 7. Results

### 7.1. Overall Description of the Included and Excluded Studies

**Figure 1:**
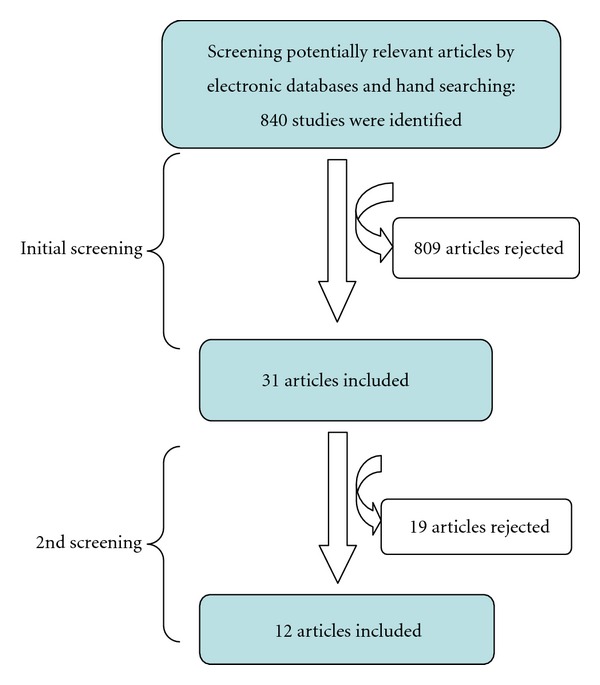
Flow diagram of the study selection process.

After the initial screening of identified papers for the present study, there were 840 potentially relevant studies found by searching either the electronic databases (PUBMED) or by hand searching papers from the literature. Unpublished papers were found by searching both the electronic databases or by hand searching. 31 studies were regarded as relevant for this study while 809 studies were excluded. The 31 selected studies were grouped into four categories in terms of the methodology identifying DH/RS: (1) clinical test (20 studies∗) (2) questionnaire based (7 studies) (3) combined questionnaire and clinical (3 studies) (4) review paper (1 study), and (∗1 study was a combined clinical/SEM in vitro study). Following evaluation of 31 studies, 19 studies were excluded (Table 1), and 12 studies were included (Table 2). Of these 12 studies a further subdivision of the category (included studies) was agreed by the two reviewers: (1) non-surgical therapy, (2) surgical therapy, and (3) a combination of the two. The flow diagram (Figure 1) of the selection procedure is illustrated below. 

### 7.2. Excluded Studies

There were five studies excluded from the present studies because neither the methodology for DH/RS assessment nor previously reported sensitivity data was described by the investigators [14, 25, 29, 33, 37]. One study was excluded for lack of any recorded objective clinical methodology evaluating DH/RS [35]. Five studies were excluded as they were reported in an abstract [26, 28, 30, 31, 36]. Further five studies were excluded as the investigators did not report any previous DH/RS history before periodontal therapy [11, 23, 32, 34, 38]. The Haugen and Johansen study [25] was excluded as this was a single case report study focusing mainly on the SEM characteristics of extracted teeth. The systematic review by von Troil et al. [8] and the clinical reports by Matthews and McCulloch [27] and Kontturi-Närhi[10] were also excluded from this paper following a further discussion by the two examiners (YH/DG) but were considered for comparison with the findings of the present study in the discussion section. In summary, 19 studies were excluded, and the reasons for exclusion were described as above (Table 1). 

### 7.3. Analysis of Included Studies

#### 7.3.1. Study Design

The studies included in the present review were comprised of either case reports, nonrandomized controlled studies, randomized controlled studies (RCT) (double-blind, single-blind, nonblind studies), questionnaire, systematic review, and so forth. All included studies were accepted as the study design, intervention and participants age were within the study criteria for inclusion (Table 2). There were five studies which involved using randomized controlled trials (RCT) [15, 40–43]. Two of the RCT were reported by the investigators as being of a double-blind design [27, 43]. One study was single-blind [42]. Two other studies did not specify the subtype of RCT [15, 41]. A nonrandomized controlled trial study design can be observed in six of the other included studies [9, 12, 39, 44, 45, 47]. Three studies reported in the present review used either a questionnaire-based design [44] or utilized both a questionnaire and clinical design in their studies [9, 47]. These three studies, however, did not have a control group. 

#### 7.3.2. Study Population

Most included studies were conducted in the setting of a specialist periodontal clinic unit in dental practices or university hospitals. Participants usually having a recognized periodontal condition were referred by local dentists to the university hospitals or specialist clinics. Most study participants in the included studies were healthy patients with a recognized periodontal condition, attending or being referred to periodontal clinics in universities in different countries. As for the gender distribution, most clinical studies enrolled almost equal numbers of participants in females or males. Some studies however were not balanced or stratified for gender. For example, four studies were predominantly female [9, 15, 41, 45] whereas the other two studies were predominantly male [42, 47]. However, some studies did not mention the gender distribution [39, 40, 44]. 

#### 7.3.3. Age Range of Participants

Although the age distributions vary widely from study to study, most participants in the included studies were adult (>18 years old). Five studies reported on the mean of age of participants [12, 40, 41, 46, 47] whereas Uchida et al. [40] failed to report any information on the age of the participants. Two studies were very similar with reported mean age in the mid 40 years old range [12, 15]. This omission may be significant if the age variable is a factor for the reported differences of DH between studies. The differences between the reported mean ranges of published studies may also be due to the different populations recruited for these studies.

#### 7.3.4. Study Duration

Most studies evaluating the effects of postperiodontal therapy on DH/RS were short term in nature lasting no longer than three months. For instance, two studies [44, 46] investigated the very early pain response following the “wearing off” of the local anesthesia given during periodontal therapy. Three studies followed up their patients for less than one month monitoring the change of sensitivity [15, 42, 47]. Five studies followed up the outcome of sensitivity for 6 range 8 weeks [9, 12, 39, 40, 43], whereas two studies followed up the outcome of sensitivity for three months [41, 45]. 

#### 7.3.5. Statistics Power Calculation

None of the included studies for this paper described or reported any statistical power calculation prior to the commencement of their studies, and given the relatively small sizes of their study populations it may be that a Type II error may have occurred which could in turn have affected their results particularly when comparing different surgical or intervention procedures (Table 3). 

#### 7.3.6. Randomization and Allocation Concealment

Of the 12 included studies in the present study only 5 [15, 40–43] reported on any details of randomization and/or allocation concealment.

#### 7.3.7. Consideration of Withdrawals and Dropouts

Withdrawals and dropouts were reported in only 4 of the included studies; for example, Fischer et al. [9] (2 out of 13 subjects), Tammaro et al. [12] (14 out of 49 subjects), Vaitkevičienė et al. [15] (five out of 67 subjects), and Gong et al. [47] (7 out of 45 subjects). None of those studies however mentioned the reasons for dropout (Table 3). 

## 8. Data Analysis

There were variations in the reporting of prevalence data, incidence of DH/RS, intensity of DH/RS, and changes in intensity of DH/RS over the duration of the study as reported by the investigators of the included studies. As a result of this variation in the reporting of the data either in average percentages over time, category scales, or differences in VAS assessment, it was difficult to compare data across the studies.

### 8.1. Previous DH History of Sensitivity

A previous DH history of sensitivity when reported by investigators in the included studies was reported in the form of either percentages and/or mean scales (VAS/Heft-Parker pain scale) and form the baseline data. Prevalence of DH/RS before periodontal treatment was reported by several investigators as being between 0% and 30.6% based on subject reporting [9, 12, 45–47] or between 21% and 69.9% based on assessment of the test teeth [15, 39]. Pre-treatment pain intensity data for DH/RS was reported by several investigators to be in a range of none to moderate discomfort [12, 15, 39–47].

### 8.2. Types of Treatment Intervention

Six included studies were based on a nonsurgical therapy intervention including oral hygiene instruction, supragingival scaling and subgingival scaling and root planning [9, 12, 42, 44, 45, 47]. The Grant et al. study compared two inserts of scalers (metal and plastic) to determine which scaler insert would cause less pain following scaling. The administration of a local anesthesia during treatment was reported in two studies [9, 44] and DH/RS was subsequently assessed after 1 week, and 3 range 4 hours after local anesthesia was given respectively. Furthermore, in the study of Pilhstrom et al. [44], 23% patients reported using analgesic medication to relieve postprocedural pain; however, the duration of taking medicine was unknown. Four of the included studies involving surgical therapy, which included either gingivectomy, open flap debridement, apical positioned flap, modified Widman Flap (±osseous contouring, bone grafting, and papilla preservation flap (±Emdogain) procedures [15, 39, 40, 43]. Only Wang et al. [43] provided any information on analgesic medication given to patients to relieve postoperative pain. Two studies combined both nonsurgical and surgical therapy intervention procedures [41, 46]. In the study of Wallace and Bissada [41], nonsurgical therapy included SRP and surgical therapy involving a modified Widman flap or apical positioned thickness flap (with osseous contouring and decalcified freeze dry bone grafting) procedures. In the study by C. F. Canakçi and V. Canakçi [46] both nonsurgical therapy (including SRP) and surgicaltherapy (modified Widman flap, open flap, or gingivectomy) were compared. However, none of these studies reported providing any local anesthesia for the nonsurgical group. No postoperative analgesic was reported as being provided or taken by patients in these studies. Four of the included studies were, however designed for testing desensitizing products in addition to the periodontal therapy procedure provided by the Investigators [15, 40, 43, 45]. 

### 8.3. Clinical Methodology Used to Assess DH/RS

 The most commonly reported method used for evaluating DH/RS by investigators in this paper was a cold air stimulus (dental air syringe). Other methods included thermal testing (cold/hot), explorer probe, electric pulp test, and questionnaire reporting (Table 4). Most studies reported using two or three methods to evaluate DH/RS [9, 12, 39–43, 45, 47]; however two studies used only an air stimulus method [15, 46] and one study only used a questionnaire [44] (Table 4).

### 8.4. Prevalence of DH/RS following Periodontal Therapy

When considering studies for inclusion in the present study, it was observed that there were a number of differences in the reporting of DH/RS, due in part to variables such as clinical methodology, study design, and duration of followup. For example, the Pilhstrom et al. [44] study lasted for less than one day and recorded DH/RS following scaling procedures by a questionnaire whereas other included studies reported over longer periods using clinical evaluation (Table 4). The incidence of DH/RS reported in some of the included studies, comparing before and after therapy intervention, ranged from 23% to 80.4%, depending on the duration of the study and the type of therapy that was provided [9, 12, 39, 45–47]. There were inconsistencies in the recording of DH/RS data as in the Pilhstrom et al. [44] study which makes comparison between the included studies very difficult. Due to variations in the study duration of the included articles, it was observed that the recorded prevalence of DH/RS peaked at different times. For example most of the short-term clinical studies reported that the prevalence peaked about one week following periodontal therapy, ranging from 36.8% to 100% after which the prevalence subsequently decreased [9, 12, 39, 40, 45, 47]. Other studies only reported on the final prevalence data without necessarily specifying any change in prevalence/incidence over time [44, 46]. For example, one study [9] comparing the influence on sensitivity resulted from either supragingival and subgingival scaling and reported that the prevalence following subgingival scaling was greater than supragingival scaling as assessed by both clinical testing and questionnaire. C. F. Canakçi and V. Canakçi [46] also compared the influence of both nonsurgical and surgical therapy and reported that there was a greater prevalence following surgical therapy than in nonsurgical therapy; however, it should be noted that this was only an observation 24 hours after therapy. 

### 8.5. The Intensity of Sensitivity following Periodontal Therapy

In the present study, the assessment of any intensity from DH/RS following periodontal therapy in most of the included studies was rated by visual analogue scales (e.g., VAS: 0–3/0–10 cm/: 0–100 mm scale; Heft-Parker 0–170 mm scale) or verbal rating (faint/weak/mild/moderate/severe, etc.) scales. Any change in the intensity of reported DH/RS between pretherapy (baseline) and posttherapy (result) can only be compared in some of the included studies [12, 39, 41, 43, 45–47]. The reported onset of DH/RS as reported in the included clinical studies generally peaked 2.8 hours after therapy up to 2 weeks following periodontal therapy [39, 44, 47]. The change of intensity from the baseline to peak time also fluctuated significantly in the included short-term studies, from 1 day to 3 months following therapy. Most of the included studies reported that the intensity of DH/RS was mild to moderate in nature and following an initial increase in severity returned to baseline values over time. However due to the differences in the reporting of DH/RS by the various investigators who used different methodology to assess the pain response it was difficult to compare the results from these studies.

### 8.6. Calibration of Indices and Examiner Training

No training or calibration for DH/RS methodology was reported in the 12 included studies. The study by C. F. Canakçi and V. Canakçi [46] reported having a trained and calibrated examiner (CFC) who only determined each patient's clinical probing depth, probing clinical attachment level (CAL), and dental plaque and bleeding on probing (BOP). These investigators however failed to report any kappa values or reproducibility data from the calibration exercise. 

## 9. Discussion

 Dentine hypersensitivity (DH) is a recognized clinical condition that has been reported to affect the adult population at various stages of their life. It is essentially a diagnosis of exclusion [2]. Periodontal therapy in the form of nonsurgical and surgical procedures are common procedures in both dental and periodontal clinics, and patients often report experiencing discomfort (in the form of DH/RS) immediately following these procedures or once the local anaesthetic has worn off [44]. However, according to several investigators data on the impact on the quality of life of those who suffer from DH/RS following these procedures is somewhat lacking. According to von Troil et al. [8] there are limited data available in terms of both prevalence, and intensity of DH/RS following periodontal therapy (such as scaling root surface, debridement and surgical procedures). 

The previous systematic review by von Troil et al. [8] examined the literature on the prevalence/incidence and severity of DH/RS; however, this paper only reported on two included studies [9, 12]; neither of these studies were randomized and one study had no control group [48]. Furthermore both of these studies measured DH/RS by different methodologies, for example, (1) by mechanical force (probe) or air stimulation and (2) by subjective patient response in the form of VAS or using a different study design (e.g., split mouth design in Tammaro et al. [12], or the duration of followup was different (4 weeks versus 8 weeks).The present study included 12 studies following an extensive review of the published literature description up to 31st December 2009. The reasons for the differences in the number of included studies in the present study and the von Troil et al. [8] review are based on a number of factors. For example, since 2002 there have been a number of studies that have been published and have been included in the present study if they fulfilled the inclusion criteria for the review. Secondly, a number of studies excluded by von Troil et al. [8], but included in the present study were due to differences in the inclusion criteria between the two reviews. The von Troil et al. review [8] included only studies that had at least one follow-up time point at least 12 months following completion of the treatment phase whereas in the present study both short- and long-term studies were included for review as both nonsurgical and surgical studies were investigated to determine any causal relation between DH/RS and periodontal therapy. Short-term studies that were excluded by von Troil et al. [8] but included in the present study were the studies by Wallace and Bissida [41] and Grant et al. [42] following agreement between the Authors. A detailed analysis of differences between the included studies in the present study has highlighted a number of problems that may prevent an overall understanding of the extent and severity of DH/RS following periodontal therapy, and these will be now addressed in the following section. It is generally accepted that a double-blind, randomized, and parallel clinical trial (RCT) is on the top of hierarchy of levels of evidence when considering the quality of a clinical study [49]. In the present study there were five studies involved in using a randomized controlled trial design (RCT) [15, 40–43]. Two of the RCTs were reported by investigators as being of a double-blind design [40, 43]. One study was single-blind [42]. Two other studies did not specify the subtype of RCT [15, 41]. A nonrandomized controlled trial study design can be observed in six of the other included studies [9, 12, 39, 44, 45, 47]. Three studies reported in the present review used either a questionnaire-based design [44] or utilized both a questionnaire and a clinical design in their studies [9, 47]. These three studies; however, did not have a control group. 

According to (consolidated standards of reporting trials) CONSORT [50] guidelines, a generated allocation schedule should be implemented by using allocation concealment, since allocation concealment is a critical process that prevents foreknowledge of treatment and thus shields those who enroll participants from being influenced by this knowledge. However of the 12 included studies in the present study only two reported on details of randomization and/or allocation concealment. For example, Grant et al. [42] and Wang et al. [43] reported using coin flip for allocation purposes. Other included studies did not report or describe any steps that were taken to conceal the allocation sequence when the various interventions were assigned. It should be noted however the majority of studies in this paper were non-randomized controlled trials [9, 12, 39, 44, 45, 47] in which a time series analysis was used that but no control/placebo was allocated in the treatments/interventions. Generally speaking, this design isused whenrandomizationis impossible, impractical, or unethical, it enables the investigator to make some generalizationsaboutthe population in the study and it is considered efficient inlongitudinalresearch. However, this deficiency in randomization of treatments/interventions makes it harder to rule out anyconfounding variablesthat may occur, and this may introduce new threats tointernal validity of the data. Since randomization is absent from the study design, some knowledge about the data can be approximated although subsequent conclusions ofcausalrelationships are difficult to determine. Therefore, in this aspect, the strength of evidence of this present study is not very robust due to the various designs in the included studies. 

A further problem regarding the included studies in this paper described or reported a statistical power calculation prior to the commencement of their studies and given the relatively small sizes of their study populations it may be that a Type II error may have occurred which could in turn have affected their results particularly when comparing different surgical or nonsurgical intervention procedures (Table 3). Furthermore from a statistical analysis point of view, published DH/RS efficacy studies usually consider the subject rather than the tooth as the experimental unit although subjects may have multiple sites which can be scored at each evaluation visit [51]. Five of the included studies, however, reported data for the test tooth rather than for the subject [15, 40, 43, 45]. Most of the included studies were conducted in the setting of a specialist periodontal clinic unit in dental practices or university hospitals. It was not clear from some of these studies whether all the participants had a similar diagnosis of periodontal disease, and it may be that some of the participants did not have any established periodontal disease. This observation made it problematic when trying to assess whether that is a causal link between the prevalence of DH/RS and periodontal procedures in periodontally involved patients. A further problem observed in the present study was the question of gender balance and while most of the clinical studies enrolled almost equal numbers of participants in females or males, some of the studies were not balanced or stratified for gender. For example, four studies were predominantly female [9, 15, 41, 45] whereas the other two studies were predominantly male [42, 43]. Three studies did not mention the gender distribution [39, 40, 44]. The reported age range of the participants from the included studies, varied from study to study although mean age values were not reported in every study. The range of mean ages in this paper was between 30 and 63.4 years of age [12, 15, 46, 47]. This is similar to the age range reported in DH/RS previous studies although the peak prevalence in an age may vary depending on the type of population being assessed [6, 20, 40, 52]. Most of studies in this paper evaluating DH/RS following periodontal therapy and were short term in nature, lasting no longer than three months and this was in contrast to von Troil et al. [8] study that excluded short-term studies. Generally speaking some of the included studies were not DH/RS efficacy studies, and as such this was not the focus of their study however while it is important to determine the actual prevalence/incidence of DH/RS following periodontal procedures, it is essential to utilize recognized methodology and study designs that can accurately report on subsequent outcomes. The recommendations by Holland [51] are useful in that they suggest an eight-week study duration based on established clinical methodology which would be a suitable time period. It should be noted that these recommendations relate to DH/RS efficacy studies, but nevertheless they would be a useful addition to the type of study included in this paper process. One major problem that may occur in a clinical study is that of patient compliance, and this may be for a number of reasons; for example, if the intervention or treatment in a study is perceived by the participant to be uncomfortable or unpleasant, this may in turn dissuade them in complying with the conditions of the study. This is particularly true in studies of a long duration but can also affect studies of a short-term nature, and sometime this may be due to relative straightforward reasons such as moving away from the area, sickness, and pregnancy) but of obvious concern to investigators and the regulatory authorities would be if the intervention caused a serious reaction in an individual. It is therefore important to record not only the number of dropouts from a study but also the reasons for dropping out as this may have a profound effect on the data to be analyzed [51]. One way of resolving this problem is to include data of these participants as if they were still in the study, this is called the intention-to-treat analysis. Withdrawals and dropouts were reported in only 4 of the included studies, for example Fischer et al. [9] (2 out of 13 subjects), Tammaro et al. [12](14 out of 49 subjects), Vaitkevičienė et al. [15] (five out of 67 subjects), and Gong et al. [47] (7 out of 45 subjects). None of those studies however mentioned the reasons for dropout (Table 3). 

Prior to any study it is essential for those assessing clinical outcomes to have both adequate training and be calibrated in the indices that they will be assessing throughout the study. From the present review of the included studies it was observed that only one article by C. F. Canakçi and V. Canakçi [46] reported an examiner calibration. However this particular calibration only determined the patient's clinical probing depth, probing clinical attachment level, dental plaque, and bleeding scores, but this calibration did not involve any recognized methodology in identifying DH/RS. A further observation from these included studies is that it was uncertain whether the same examiner assessing for DH/RS was the same clinician performing the periodontal therapy for the participant. It is therefore possible that unless this was accounted for in these studies, there is a potential risk of bias which may subsequently affect the results of the study. It is also clear from the included studies that a number of assessment tools were employed in the study such as an air stimulus, explorer probe, or a thermal stimulus. Several investigators have recommended that at least two hydrodynamic stimuli should be used in clinical testing as a single method may underestimate the true prevalence of DH/RS [51, 53, 54]. Two studies in this paper only used an air stimulus for identifying DH/RS [15, 46]. The sequence of applying the various stimuli is also important, and it has been previously recommended that the least invasive stimulus (e.g., an explorer probe) should be used before an air or thermal stimulus [53]. One study by Fischer et al. [9] reported using a probe stimulus prior to an air stimulus to assess DH/RS; however, most of the other studies failed to mention the sequence of testing which may have had a subsequent effect on the results. Most studies failed to record any details regarding the use of their assessment methodology in their papers, for example, the probing pressure, only one study by Tammaro et al. [12] (0.45N 45.9 g) or the air syringe [12, 15, 41, 42, 45, 46], and this may also have introduced further difficulties when trying to evaluate the results from the studies. Other investigators used alternative clinical methodology such as water [43] and electrical assessment [9, 41, 43] (Table 4) although the use of this type of stimuli in DH/RS has been criticized for a number of reasons [52, 55, 56]. One study by Wolff et al. [36] which was excluded for the purpose of the present review as it was only an abstract is worth mentioning here since the investigators utilized both tactile (Yeaple probe: a controlled force probe) and thermal (dental air syringe) stimuli together with a subjective evaluation (VAS). This small pilot study with only 9 subjects (24 teeth) characterized the incidence of DH/RS following periodontal surgery over a six-week period. The investigators assessed DH/RS prior to the surgery, baseline (postsurgery) and over 6 weeks and reported that when assessed by standardized DH methodology the incidence of DH/RS returned to the pre-surgical levels between 4 and 6 weeks. Although in abstract form with a very small sample size, it was clear from the available details that the study is following a recommended protocol based on the guidelines for evaluating DH, and it would appear to be the basis of a model for evaluating the incidence and or prevalence following periodontal surgery although the duration of such a study would have to be extended if desensitizing products were to be evaluated on the participants [51].

Although the importance of standardized methodology is essential in the assessment of DH/R,S it should be noted that pain measurement also requires subjective evaluation and as such should also be included in the assessment of DH/RS [54]. Traditional methods may provide an indirect assessment of pain by numerical estimates of detection, pain, or tolerance thresholds. Contemporary methods can provide a direct assessment of the pain strength by the expression of pain in units of the subjective intensity. Both methods of assessing DH/RS are valid if they are utilized in a well-conducted clinical study [51, 53, 54]. Three studies used a questionnaire to assess DH/RS following scaling [9, 44, 47]. Pilhstrom et al. [44] provided participants with a take-home pain assessment form 3 to 4 hours following scaling procedures in order to assess the pain response. Fischer et al. [9] provided a questionnaire to patients at each clinical interval whereas Gong et al. [47] utilized both questionnaire and clinical testing at each clinical session, prescaling and root planning, at 1 week and 4 weeks, respectively after treatment for an overall assessment of DH/RS on a daily basis. Measurement of the pain response is inherently difficult as it is highly subjective, and its perception and subsequent pain may differ widely among individuals [57–60]. Furthermore it is recognized that there are profound placebo and nonplacebo effects, regression to mode or mean, occurring during clinical studies, and this may also have a profound effect on the subsequent results [61]. A further problem when evaluating the true prevalence or incidence of DH/RS from the included studies was the effect of the local anaesthetic provided during the treatment session, together with the intervention medication that may have been taken after the completion of treatment. For example the study by Pilhstrom et al. [44] reported that the questionnaires were given out to the participants 3 hours after treatment, and it is possible that the recovery from the local anesthesia may have varied and impacted on the participant's perception of pain. Analgesia medication may have also been taken by the participants, and this may have interfered with the participants' pain and impacted on the subsequent prevalence or incidence of DH/RS [43, 44]. 

Most studies evaluating the effects of postperiodontal therapy on DH/RS were short term in nature lasting no longer than three months. For instance, two studies [44, 46] investigated the very early pain response following the “wearing off” of the local anesthesia given during periodontal therapy. Three studies followed up their patients for less than one month monitoring the change of sensitivity [15, 42, 47]. Five studies followed up the outcome of sensitivity for 6 range 8 weeks [9, 12, 39, 40, 43] whereas two studies followed up the outcome of sensitivity for three months [41, 45]. The reported duration of pain in the included studies lasted from a few days to a few years depending on the type of studies reported (questionnaire or clinical outcomes) which is consistent with other studies [10, 23, 30]. In general, the reported pain duration was shorter in the clinical studies (less than 2 months). The reported onset of DH/RS, symptoms fluctuated significantly over time and depending on the type and duration of the study DH/RS peaked at 2.8 hours [44], and up to 1 range 3 weeks following periodontal therapy [12, 15, 39, 43]. Several studies have reported that periodontal treatment (non-surgical and surgical) is frequently associated with pain which is generally mild in nature [27, 38, 42, 57–60]. The results from the present study were similar to those reported by von Troil et al. [8] which is not surprising considered both reviews incorporated studies that provide similar data on the prevalence of DH/RS following periodontal therapy, the reduction of the gingival protective barrier as a result of the surgical excision of tissue which subsequently exposes the root surfaces, whereas scaling and root-planing procedures (SRP) may remove 20–50 micrometers of cementum thus exposing the dentinal tubules to external stimuli [39, 41]. An included study by C. F. Canakçi and V. Canakçi [46] compared the postoperative pain and DH/RS from different periodontal procedures. In general, these investigators reported that postoperative pain and postoperative sensitivity were significantly higher in surgical procedures. In particular, a flap design with osseous resection resulted in the highest degree of discomfort which may be as a result of a time-consuming procedure together with and exposure of bone. Participants who experienced surgical procedures appeared to be more likely to have increased discomfort from DH/RS (1.3 and 1.4X) than non-surgical procedures. 

The reported prevalence of DH/RS following surgical therapy was 76.8% range 80.4% after 1 day [46], 36.8% after 1 week, 33.4% after 2 weeks, 29.6% after 4 weeks, and 21.7% after 8 weeks [39] whereas the prevalence following nonsurgical therapy was reported to be between 62.5% and 90% after 1 day of nonsurgical intervention [44, 46], 52.6% and 55% after 1week of nonsurgical therapy [9, 12, 45, 47].

The increase of DH/RS prevalence in studies that compared pre- and posttherapy intervention ranged from 23% to 80.4%, depending on the type of therapy provided [9, 12, 39, 45–47]. The observation that surgical procedures aggravate DH/RS as compared with nonsurgical procedures would appear to be supported by most of the studies included in the present review. For example, both Wallace and Bissada [41] and C. F. Canakçi and V. Canakçi [46] reported that DH/RS appeared to increase to mild-to-moderate intensity levels following periodontal therapy. One should note however that the study by C. F. Canakçi and V. Canakçi [46] was only up to 24 hours. According to Tammaro et al. [12] sub-gingival scaling procedures caused more sensitivity than supragingival scaling procedures. This observation was also supported by Fischer et al. [9]. 

It was difficult however to compare the various treatment procedures (nonsurgical/surgical, type of surgical procedure, etc.) and their subsequent effect on DH/RS. This is due in part to the differences between all the studies included in this present review, whether it is the type of study, the duration of the study, the type of treatment intervention, the methodologies employed, or the manner in which the results were recorded (in terms of percentages or pain categories, etc.); all have impact in the attempt to analyze the data. As a consequence of the heterogeneous nature of the data reported in the 12 included studies either in terms of different study designs and variations in the analysis of the pain response (average percentages over time, category scales, differences in VAS assessment, etc.) it was decided not to conduct any further statistical analysis of the data [8]. The results from the present study were similar to those previously reported by von Troil et al. [8], in terms of reported prevalence of DH/RS following periodontal procedures which is not surprising, considering that both reviews incorporated studies with the same data. However it was evident that despite the previous recommendations of von Troil et al. [8] and of Holland [51], none of the studies include in the present review used recognized or standardized methodology for assessing DH/RS apart from Tammaro et al. study [12] (note that this study was included in the von Troil et al. review [8]). According to von Troil et al. [8] the prevalence of DH/RS before any treatment was between 9 and 23% and 54 and 55% following the allocated periodontal therapy (nonsurgical procedures) whereas in the present study the reported prevalence for DH/RS (following nonsurgical intervention) was between 62.5% and 90% treatment [44, 46] after 1 day which subsequently decreased to approximately 52.6% range 55% after one week [9, 12, 45, 47]. In the present review the prevalence of DH/RS following surgical therapy ranged from 76.8% to 80.4% after 1 day following treatment [46], and subsequently decreased over time to 36.8% after 1 week, 33.4% after 2 weeks, 29.6% after 4 weeks, and 21.7% after 8 weeks [39]. However in both the von Troil et al. [8] review and in the present study there was a lack of standardized data relating to any patient-based (subjective) (e.g., VAS) evaluation of DH/RS following periodontal procedures. 

## 10. Conclusions

It is evident from reviewing the included studies that patients may suffer from mild discomfort following periodontal procedures although both the prevalence and intensity of DH/RS may vary depending on the duration and the type of procedure involved. Most of the studies included in this paper would tend to suggest that DH/RS may be relatively mild/moderate in nature and transient in duration. Currently there appears to be limited data on the effect of DH/RS following periodontal procedures on the quality of life of patients, and it is recommended that both short- and long-term studies should be implemented to determine whether there is a major impact on life style following these procedures. 

## Figures and Tables

**Table 1:** Characteristics of excluded studies (reasons for exclusion).

Excluded studies	Reasons for exclusion
Haugen and Johansen [25]	(1) Single case report, (2) unreported baseline data, (3) unreported method assessing DH/RS, and (4) SEM study
Sim and Han [26]	Unreported baseline data; abstract only
Matthews and McCulloch [27]	Included participants under 18 years old
Kontturi-Närhi [10]	Not all participants in clinical test study were reported having periodontal treatment
Kiyonobu et al. [28]	Unreported baseline data; abstract only
Chabanski [11]	Unreported baseline data
Zetterström [29]	Unreported baseline data; unreported method assessing DH/RS
Kontturi-Närhi and Närhi [30]	Unreported baseline data; abstract only
Tamminen et al. [31]	Abstract only
Gillam et al. [32]	Unreported baseline data
Heard et al. [33]	Unreported baseline data
Gillam et al. [23]	Unreported baseline data
Clayton et al. [34]	Unreported baseline data
Fardal et al. [35]	Unreported method assessing DH/RS
von Troil et al. [8]	Systematic review
Wolff et al. [36]	Abstract only
Froum et al. [37]	Unreported baseline data
Tonetti et al. [14]	Unreported baseline data
Al-Hamdan [38]	Unreported baseline data

**Table 2:** Characteristics of included studies (reasons for inclusion).

Study	Reason for inclusion
Nishida et al. [39]	The study design, intervention, and participants age were within the study criteria for inclusion
Uchida et al. [40]	The study design, intervention, and participants age were within the study criteria for inclusion
Wallace and Bissada [41]	The study design, intervention, and participants age were within the study criteria for inclusion
Fischer et al. [9]	The study design, intervention, and participants age were within the study criteria for inclusion
Grant et al. [42]	The study design, intervention, and participants age were within the study criteria for inclusion
Wang et al. [43]	The study design, intervention, and participants age were within the study criteria for inclusion
Pihlstrom et al. [44]	The study design, intervention, and participants age were within the study criteria for inclusion
Tammaro et al. [12]	The study design, intervention, and participants age were within the study criteria for inclusion
Xu and Yang [45]	The study design, intervention, and participants age were within the study criteria for inclusion
Vaitkevičienė et al. [15]	The study design, intervention, and participants age were within the study criteria for inclusion
C. F. Canakçi and V. Canakçi [46]	The study design, intervention, and participants age were within the study criteria for inclusion
Gong et al. [47]	The study design, intervention, and participants age were within the study criteria for inclusion

**Table 3:** Number of participants completing study and number of teeth assessed during the study.

Author	The numbers of participants	Statistics calculated
Nishida et al. [39]	54 subjects (290 teeth)	Unreported
Uchida et al. [40]	60 subjects (249 teeth)	Unreported
Wallace and Bissada [41]	10 subjects	Unreported
Fischer et al. [9]	13 subjects (2 dropouts reason not given)	Unreported
Grant et al. [42]	23 subjects	Unreported
Wang et al. [43]	25 patients	Unreported
Pihlstrom et al. [44]	52 subjects	Unreported
Tammaro et al. [12]	49 subjects (14 dropouts reason not given)	Unreported
Xu and Yang [45]	52 subjects (1453 teeth)	Unreported
Vaitkevičienė et al. [15]	67 subjects (5 dropouts reason not given)	Unreported
C. F. Canakçi and V. Canakçi [46]	56 subjects	Unreported
Gong et al. [47]	45 subjects (7 dropouts reason not given)	Unreported

**Table 4:** Clinical methodology used to assess DH/RS in the included studies.

Author	Probe test	Air	Thermal	EPT	Questionnaire
Nishida et al. [39]	**+**	**+**	**+**		
Uchida et al. [40]	**+**	**+**	**+**		
Wallace and Bissada [41]		**+**	**+**	**+**	
Fischer et al. [9]	**+**	**+**		**+**	**+**
Grant et al. [42]	**+**	**+**			
Wang et al. [43]	**+**		**+**	**+**	
Pihlstrom et al. [44]					**+**
Tammaro et al. [12]	**+**	**+**			
Xu and Yang [45]	**+**	**+**	**+**		
Vaitkevičienė et al. [15]		**+**			
C. F. Canakçi and V. Canakçi [46]		**+**			
Gong et al. [47]		**+**			**+**

“+” represents the method included in each included study to identify DH/RS.
